# Research Advances and Perspectives on Early Flowering Traits in Cucumber

**DOI:** 10.3390/plants14081158

**Published:** 2025-04-08

**Authors:** Meidi Zhang, Ming Ma, Hong Lang, Mingliang Jiang

**Affiliations:** School of Agriculture, Jilin Agricultural Science and Technology University, Jilin 132101, China; zhangmeidi@jlnku.edu.cn (M.Z.); maming_jlnku@163.com (M.M.)

**Keywords:** cucumber, early flowering, research advances, challenges, perspectives

## Abstract

Early flowering refers to the phenomenon in which the first flower appears in fewer days than normal, regardless of the sex of the flower. It is a significant feature impacting the early maturity and economic yield of cucumbers. The early flowering trait of cucumber is influenced by several factors. Considering its heritability, technologies such as whole-genome sequencing, genetic modification, bioinformatics analysis, quantitative trait locus (QTL) mapping, molecular marker-assisted selection, and gene editing are widely used to explore the regulatory genes and molecular mechanisms of the early flowering trait in cucumbers. This review aimed to summarize the factors, QTL mapping, molecular regulation mechanisms, and omics analysis related to early flowering traits in cucumbers. This review contributes theoretical insights to support both cucumber breeding for early flowering and fundamental research on early flowering traits.

## 1. Introduction

Cucumber (*Cucumis sativus* L., 2n = 14) belongs to the Cucurbitaceae family and *Cucumis* genus; it is an important vegetable crop with the largest planting area and yield among vegetables and plays a vital role in the annual production and supply of vegetables [1,2]. The wild type of cucumber, *C. sativus* var. *hardwickei*, was originated in India [3]. Cucumbers have adapted to various climatic conditions due to natural and artificial selection. The bitter taste of cucumber fruit disappears, the morphological diversity and adaptability change, and both yield and quality are improved during domestication. Additionally, the sensitivity of cucumber plants to extended photoperiods has changed due to domestication. Different types of cucumbers can bloom rapidly only under suitable photoperiod conditions. However, the molecular processes of cucumber domestication remain unclear [4]. The phylogenetic analysis shows that cucumbers are classified into four geographic groups: the wild Indochina group, the semi-wild Xishuangbanna group, and two cultivated cucumber groups (Eurasia and East Asia) [5].

Yunnan, China, is considered the secondary center of the origin of cucumbers. Cucumbers have been planted on a large scale for many years; however, their planting areas and yields continue to increase steadily worldwide. The total area of cucumber cultivation worldwide was 2.25 million hectares in 2020, yielding 9.035 million tons. China accounted for 56.4% (1.27 million hectares) of the global cucumber production area and 81.2% (73.36 million tons) of the global cucumber production [6]. As a fruit and a vegetable, early maturing cucumber offers high economic benefits; therefore, early maturity has become a crucial focus for efficient and high-yield breeding [7]. Cucumber breeders have also shifted their focus to developing early maturing and high-yielding varieties [8,9]. Previous studies have extensively investigated the agronomic traits related to the early maturity of cucumbers. These traits related to the early maturity of cucumbers mainly include the position of the first flowering node, the position of the first female flower, and the number of days from sowing to the opening of the first female flower during the initial flowering period [10]. Cucumber domestication has significantly improved yield, quality, and photoperiod adaptation. However, the underlying molecular mechanisms need complete elucidation. The increasing global demand for cucumbers, particularly in China as the dominant producer, has led to breeding efforts focused on early maturing varieties with enhanced economic value.

Early flowering refers to the phenomenon in which the first flower appears in fewer days than normal, regardless of the sex of the flower; it is a significant feature impacting the early maturity and economic yield of cucumbers [7,11,12]. The flowering transformation is triggered by multiple factors combining endogenous genetic pathways with environmental stimuli [13,14,15]. Early flowering can improve the economic benefits of crops; hence, the mechanisms of flowering in many crops have been explored, and early flowering is a crucial breeding goal [16]. Various pathways influencing flowering have been reported in *Arabidopsis* [14]. These include endogenous (autonomous, gibberellin, circadian rhythm, and age) and environmental (vernalization, environmental temperature, and photoperiod) factors [17,18,19,20]. In addition, factors that can cause early flowering have been detected in crops such as tomatoes [16], rice [21], wheat [22], Chinese cabbage [23], and cassava [24]. These findings indicate that early flowering is governed by conserved genetic pathways across diverse plant species while exhibiting species-specific regulatory adaptations. Understanding these shared and unique mechanisms in cucumbers may be crucial for developing targeted breeding strategies to optimize flowering time and enhance crop productivity.

The data on germplasm resources obtained through physiological and molecular investigations on cucumbers are limited. This review summarizes the environmental factors, genetic QTL mapping, molecular mechanisms, and omics research related to the early flowering of cucumbers. It has significant application value for shortening the growth period of cucumbers, balancing and optimizing the industrial demand, and increasing cucumber yields.

## 2. Factors Influencing Cucumber Early Flowering Traits

### 2.1. Light

Light is the primary driver of plant photosynthesis and one of the most important environmental cues to regulate plant growth and development [25,26]. Both the length of the photoperiod and the intensity of light can impact the flowering time of plants. Long-day (LD) plants flower when the sunshine duration exceeds the critical length, whereas short-day (SD) plants flower when the sunshine duration is shorter than the critical length. The flowering of day-neutral plants is independent of the length of daylight hours [13]. *Arabidopsis thaliana* is an LD plant, with LD accelerating flowering and SD delaying flowering [27]. Xishuangbanna (XIS) cucumber (*C. sativus* L. var. *xishuangbannesis*) is a typical SD plant requiring a certain number of SD to induce flowering [28,29]. Weak light leads to delayed flowering time, delayed peak flowering period, prolonged flowering period, and decreased flowering quality in cucumbers [12]. Plants sense light mainly via photoreceptors [30,31,32,33,34,35,36,37,38]. Among these, photosensitive pigments significantly influencing the flowering time of plants have been identified [39,40,41,42,43]. For example, Phytochrome B (PHYB) can directly or indirectly interact with the Highly Expressed Osmotically Responsive Genes 1 (HOS1), Phytochrome and Flowering Time 1 (PFT1), and Vascular Plant One-zinc Finger 1/2 (VOZ1/2) to promote or inhibit *FLOWERING LOCUS T* (*FT*) and *CONSTANS* (*CO*) expression, ultimately mediating flowering [44,45,46,47]. *CsPHYB* regulates cucumber flowering time in a photoperiod-dependent manner [48]. The changes in the genes involved in the photoperiod pathway can promote flowering in photoperiod-sensitive crops [27,29]. Previous studies found that *PHYB-deficient* mutants exhibited early flowering [49,50]. Light is essential for the normal development of plants, significantly influencing crop growth, development, and quality. The flowering of cucumbers is impacted by the length of light exposure; therefore, the early flowering process of cucumbers can be regulated through reasonable control of light exposure during production.

### 2.2. Hormones

Hormones play vital roles in plant growth and development. Flowering is regulated by several hormones, such as gibberellins (GAs), jasmonic acid (JA), abscisic acid (ABA), brassinosteroids (BRs), and salicylic acid (SA) [51,52,53,54].

#### 2.2.1. GAs

GAs have multiple physiological functions, including promoting stem elongation, breaking dormancy, promoting fruit development, affecting gender expression, delaying aging, and improving stress resistance. It has also been reported that GAs promote flowering [55,56,57,58,59,60]. A previous study showed that treating cucumbers with 100 μmol GA promoted flowering whereas the treatment of cucumbers with 100 μmol paclobutrazol (PAC) delayed flowering [48]. *CsPHYB* can delay cucumber flowering time by inhibiting the biosynthesis of GA.

#### 2.2.2. JA

JA is vital in plant growth, development, and stress resistance. It can induce the expression of early JA response genes and flower development-related proteins, thereby influencing plant flowering time [61,62,63,64,65,66]. The JA Zinc-finger protein expressed in Inflorescence Meristem (ZIM) domain protein, CsJAZ1, is linked to the Homeodomain-Leucine Zipper IV (HD-ZIP IV) protein to regulate the flowering time of male cucumber flowers [67].

#### 2.2.3. ABA

ABA mainly participates in plant stress response and growth and development regulation. It alleviates drought, salt, and low-temperature stresses; promotes seed development and dormancy; enhances root growth; and regulates stomatal closure. It also plays an essential role in plant flowering [68]. Further, it inhibits flowering [69]. It not only promotes plant dormancy but also affects flower bud differentiation [70,71]. ABA can induce the expression of the *C. sativus SHATTERPROOF* (*CsSHP*) gene, thereby influencing the formation and development of cucumber floral organs [72].

#### 2.2.4. BR and SA

BR is widely involved in plant growth, development, and stress response. It promotes cell elongation and division; regulates the development of plant stems, leaves, and roots; enhances plant drought, salt, and disease resistance; and regulates plant photosynthesis. SA mainly participates in the defense response of plants. It improves their drought resistance, stress resistance, and antioxidant effects and promotes seed germination and root development. Both BR and SA can also influence the flowering time in cucumbers [73]. Plant hormones have diverse effects on the early flowering of cucumbers. Therefore, the early flowering process of cucumbers can be modulated by regulating the concentration and synthetic pathways of hormones during production, thereby providing strong support for cucumber production.

### 2.3. Other Factors

The early flowering of cucumbers is influenced by various factors, including temperature and nutritional conditions, which can be reasonably controlled in production. The reproductive stage has the most stringent temperature requirements during plant growth and development [74]. Temperature impacts the flowering time of cucumbers; high temperatures can promote early flowering [75,76]. Moreover, treatments such as nutrient composition, inorganic fertilizers (phosphate solubilizing bacteria, PSB), and organic measures (integrated nutrient management, INM) can lead to cucumber flowering [77,78]. A previous study showed that cucumber plants treated with organic and biological fertilizers exhibited a better early flowering shape; early flowering was better than that in the control group, especially in plots treated with farmyard manure, FYM, at a concentration of 20 t ha^−1^ [79].

In conclusion, regulating cucumber flowering time involves a complex interplay of environmental factors (light and temperature), hormonal pathways (GA, JA, ABA, BR, and SA), and nutritional management. These findings indicate that the targeted manipulation of photoperiod conditions, hormone balance, and organic fertilization can effectively promote early flowering in cucumbers. Future studies should further explore the molecular mechanisms underlying the aforementioned interactions to develop more precise cultivation strategies for optimizing flowering time and yield.

## 3. Current Status of Breeding Early Flowering Cucumbers

The breeding of early flowering cucumbers is an essential direction in cucumber breeding. The growth cycle of cucumbers can be shortened and the yield and economic benefits can be improved by selecting early flowering varieties. A total of 1215 cucumber varieties were registered as non-major crop varieties in China from 2017 to 2020 [80]. Multiple breeding methods have been adopted in cucumber breeding, including hybrid, mutagenesis, molecular marker-assisted selection, and genetic engineering breeding [80]. The breeding of early flowering cucumbers also achieved significant results through the continuous efforts of breeders. For example, “Jinchun No.4” takes about 34 days from emergence to flowering [81]. “Zhongnong No.5” is a new female cucumber variety, with the first flowering occurring at two to three nodes [82]. “Kaohsiung No.3” was obtained through the hybridization of KSL009 and KSL017 inbred lines; it manifested as an early flowering variety tolerant to heat and moisture [83]. In summary, the breeding of early flowering cucumbers breeding has demonstrated significant potential in enhancing productivity and economic returns. The successful development and application of these varieties highlight the practical achievements in this field.

## 4. QTL Mapping of Cucumber Early Flowering Traits

QTL mapping is used for determining the position of quantitative trait genes on chromosomes. It is based on Morgan’s linkage inheritance law. It uses molecular markers to classify the genotype of each individual, analyzes the relationship between the genotype of each individual in the mapping population and the phenotype of the target trait, and predicts the genetic linkage and distance between QTL loci and molecular markers of the target trait [84]. Screening molecular markers is an effective means of successfully determining the relative positions of related genes. Biotechnological advances have led to the successful development of various molecular markers, such as restriction fragment length polymorphisms (RFLP), sequence-characterized amplified region (SCAR), sequence tagged sites (STS), simple sequence repeats (SSR), single primer amplified region (SPAR), single strand conformation polymorphism (SSCP), random amplified polymorphic DNA (RAPD), inter-simple sequence repeat (ISSR), sequence-related amplified polymorphism (SRAP), cleaved amplified polymorphic sequence (CAPS), and amplified fragment length polymorphism (AFLP) [85]. Employing these molecular markers is of great significance for QTL mapping and crop breeding.

Early flowering is essential for improving the early maturity and economic yield of cucumbers [7]. The early flowering of cucumbers is a typical quantitative trait [12,86]. Many studies have successfully mapped QTLs for the early flowering of cucumbers (Table 1). The gynoecious *C. sativus* var. *sativus* line “GY14” and wild *C. sativus* var. *hardwickii* (R) Alef. “PI183967” plants were used to backcross BC, S_3_, and F_2_ populations for the QTL mapping analysis of early flowering [87]. Two QTLs controlling flowering time were identified: one on linkage group 5 near the RFLP marker CsC029 and the other on linkage group 2 near the F-locus. The recombinant inbred line (RIL) and F_2_ population constructed through “G421” and “H-19” were used to conduct the genetic mapping and QTL analysis of the agronomic traits of cucumbers [88]. Four QTLs for flowering time (*ant1.1*, *ant2.1*, *ant5.1*, and *ant6.1*) were mapped to linkage groups 1, 2, 5, and 6. An F_2_ population was developed by crossing cucumber inbred lines S06 and S52 [89]. The use of 64 sequences SRAP markers indicated that the first flower node trait control gene *ffn* was located on the linkage group 9, with distances of 10.3 and 12.1 cM from the two side markers DC1EM5 and ME7EM2A, respectively. The F_9_ generation RIL populations were generated using the North China protected area-type cucumber “9930” and the European greenhouse-type cucumber “9110Gt”, and a QTL *Da1.1* located on Chr1, which is related to flowering time, was identified [10].

The phenotypic investigation and genetic analysis were conducted on the F_2_ population constructed using “9930” (late flowering) and “Muromskij” (early flowering) [86]. The results showed that the early flowering traits were quantitative traits controlled by multiple genes, and the main flowering gene in “Muromskij” was dominant. The QTL *Ef1.1*, which controls the early flowering trait, was located in the 890 kb interval of Chr1. It was speculated that *Csa1G651710*, homologous to *FT*, was a candidate gene for the cucumber early flowering QTL *Ef1.1*. A total of 124 RILs derived from the cross of the XIS cucumber with cultivated inbred lines of cucumbers (“CC3” and “SWCC8”) were used to create a linkage map. Further, *fft1.1* major-effect QTL (R^2^ = 25.8%) which was repeatedly identified in all four environments and had a highly consistent peak position on the genetic map, and *fft6.1* minor QTL (R^2^ ≈ 6%) was detected in two seasons on Chr1 and Chr6, respectively [90]. Both QTLs led to an early flowering phenotype in cucumber plants.

The F_2_ and F_2:3_ populations were constructed using the all-female cucumber line “S1000” and the strong male cucumber line “S1002” as parents, and a high-density genetic linkage map was drawn for QTL mapping [85]. A total of six QTLs were detected on Chr3, Chr5, Chr6, and Chr7: *EF3.1-1*, *EF3.1-2*, *EF5.1*, *EF6.1*, *EF6.2*, and *EF7.1*. *Csa6G382930*, *Csa7G430750*, and *Csa7G431330* were preliminarily identified as candidate genes for *EF6.1* and *EF7.1*. Another set of F_2_ and F_2:3_ populations was constructed using early flowering inbred line “WI7200” and late flowering inbred line “WI7167” of cucumber, and three QTLs controlling flowering time were identified: *FT1.1*, *FT5.1*, and *FT6.2* [91]. Among these, *FT6.2* (R^2^ = 71.9–83.3%), which was located on Chr6, played the most important role in early flowering with low photoperiod sensitivity during domestication. *FT1.1* (R^2^ = 9.1%), which was located on Chr1, was more involved in regulating the flowering time of cultivated cucumbers, with *FT5.1* (R^2^ = 4.8–16.1%) moderation influencing flowering time. The cultivated cucumber line “Gy14” and the wild cucumber line “WI7221” were used to construct RIL, F_2_, and F_2:3_ populations [29]. Two QTLs related to flowering time were identified through QTL analysis. Among these, the main QTL, *FT1.1* (R^2^ = 42.8%), played an essential role in regulating flowering, whereas the minor QTL, *FT6.3* (R^2^ = 8%), contributed to photoperiod-sensitive flowering time during domestication. One major QTL (*FT1.1*, R^2^ = 13.9–44.9%) and one minor QTL (*FT6.4*, R^2^ = 14.8%) related to flowering time were identified using segregation F_2_ and RIL populations from the cross between “WI2757” and “TL” [92].

*FT1.1* is located in the overlapping region on Chr1 [10,29,90,91,92], indicating that they may belong to the same locus. The flowering time was assessed in cucumber lines “CG5479” and “9930” with different day lengths to construct NILs; an early flowering gene *Ef1.1* was finely located [93]. RILs and F_9_ populations were constructed using “CC3” and “SWCC8” as parents, and the main QTL *DFF1.1* regulating cucumber flowering time was located on Chr1 through QTL sequence analysis [27]. Many studies have identified QTL related to early flowering in cucumbers, laying a solid foundation for subsequent mapping of early flowering genes and revealing gene functions.

Extensive QTL mapping studies have successfully identified multiple genomic regions (particularly on Chr1 and Chr6) governing early flowering traits in cucumbers. *FT1.1* has emerged as a consistently detected major-effect locus across diverse populations. Integrating high-density genetic maps and candidate gene analysis (e.g., *Csa1G651710* homologous to *FT*) has significantly advanced the understanding of the genetic architecture underlying flowering time regulation. These findings provide valuable tools for molecular marker-assisted breeding and pave the way for the functional characterization of key genes to enhance precision in improving the early flowering traits in cucumbers.

**Table 1:** QTL mapping of early flowering traits in cucumbers.

Parents	Populations	QTLs	References
GY14 * PI183967	BC, S_3_	Two chromosome regions	[87]
G421 * H-19	RIL	*ant1.1*, *ant2.1*, *ant5.1*, *ant6.1*	[88]
S06 * S52	F_2_	*ffn*	[89]
9930 * 9110Gt	F_9_	*Da1.1*	[10]
9930 * Muromskij	F_2_	*Ef1.1*	[85]
CC3 * SWCC8	RILs	*fft1.1*, *fft6.1*	[90]
S1000 * S1002	F_2_ and F_2:3_	*EF3.1-1*, *EF3.1-2*, *EF5.1*, *EF6.1*, *EF6.2*, *EF7.1*	[85]
WI7200 * WI7167	F_2_ and F_2:3_	*FT1.1*, *FT5.1*, *FT6.2*	[91]
Gy14 * WI7221	RIL, F_2_, and F_2:3_	*FT1.1*, *FT6.3*	[29]
WI2757 * TL	F_2_	*FT1.1*, *FT6.4*	[92]
CG5479 * 9930	NILs	*Ef1.1*	[93]
CC3 * SWCC8	RILs and F_9_	*DFF1.1*	[27]

The asterisk * represents hybridization.

## 5. Molecular Regulatory Mechanism of Cucumber Early Flowering Traits

Flowering is an essential indicator of the transition from vegetative to reproductive growth in higher plants [94], ensuring the production of seeds necessary for species survival [95]. It is regulated by a complex genetic pathway responding to both endogenous and environmental stimuli [13,14]. Research on *Arabidopsis* has revealed six regulatory pathways related to flowering time: vernalization, photoperiod, gibberellin, autonomy, age, and environmental temperature pathways [96,97,98], which are interconnected to form a complex regulatory network influencing flowering. Several genes related to flowering time have been identified in *A. thaliana* [96], all of which are specific flowering-related genes. These genes are named flowering integration genes [99]. They include *FT* [100], *SUPER PRESSOR OF OVEREXPRESSION OF CONSTANS 1* (*SOC1*) [101], and *LEAFY* (*LFY*) [102].

Many early flowering genes in cucumbers homologous to those in *Arabidopsis* have been identified (Table 2). For example, the *SQUAMOSA PROMOTER BINDING PROTEIN-LIKE* (*SPL*) gene was first discovered in *Antirrhinum majus* [103] and plays a critical role in various stages of plant transition from vegetative to reproductive growth [104,105]. *SPL* genes directly or indirectly regulate the flowering time in *A. thaliana* [106,107,108,109,110,111]. However, whether cucumber *CsSPL* mediates flowering remains largely unclear. Therefore, the *SQUAMOSA PROMOTER-BINDING-LIKE PROTEIN 13A* (*CsSPL13A*) gene was cloned, and CsSPL13A-OE plants were constructed. The results showed that the CsSPL13A-OE plants exhibited early flowering compared with the wild-type plants. The yeast one-hybrid and dual-luciferase reporter assays indicate that CsSPL13A protein directly binds to the promoters of *CsFT* and *β-AMYLASE* (CsBAM), up-regulates their expression, and mediates cucumber flowering [6].

*FT* belongs to the phosphatidylethanolamine-binding protein (PEBP) family and regulates the flowering time in *A. thaliana* [112,113,114,115,116]. It is an activator of flowering transition [117,118]. An early flowering QTL *Ef1.1* was mapped using the F_2_ population with “Muromskij” and “9930” hybridization [86]. The results of fine mapping and cloning showed that large genetic structural variations from *CsFT* upstream led to early flowering in cucumbers [93]. The pan-genomic analysis showed a consistent correlation between the variance of sequences located upstream of *FT* and the flowering time of the cucumber germplasm [119]. *FT* regulates SD flowering in XIS cucumbers, and the *cis*-regulation of *FT* is likely due to TE insertion [120]. The hybrid of “PI183967” and “9930” was used to cultivate and identify the photosensitive site *sp-1* under different light lengths (LD and SD) [4]. The mapping results showed that *CsFT*, which was the only member of the *FT/TSF-like* clade in the PEBP family, was a candidate gene. The overexpression of *CsFT* significantly accelerated flowering in *Arabidopsis* [121]. Therefore, *CsFT* is a crucial gene involved in cucumber photoperiod domestication.

MADS-box genes are key transcription factors involved in plant development, particularly flower development [122,123]. MADS-box genes, such as *Cucumber MADS box gene 1* (*CUM1*), *Cucumber MADS box gene 10* (*CUM10*), *Cucumber MADS box gene 26* (*CUM26*), *CsAPETALA3* (*CsAP3*), and *CsEPALLATA2* (*CsSEP2*), can affect the development of cucumber flowers; however, their functions still need confirmation [124,125,126,127]. A comprehensive analysis was conducted on 43 MADS-box genes in cucumber, which were compared with the genes in *Arabidopsis*, grape, and poplar. The expression analysis showed that 42 of 43 MADS-box members in cucumbers were expressed in various plant tissues, suggesting their varying roles in plants [128]. The MADS-box gene *CsSHP* was specifically enriched in stamens and carpels, and its overexpression resulted in early flowering in *Arabidopsis*. A yeast two-hybrid (Y2H) assay result showed the interaction of CsSHP with CsSEPs [72]. Moreover, the ectopic overexpression of *CsMADS02* and *CsMADS08* caused earlier flowering in *Arabidopsis* [129,130,131].

Branched-chain amino acids and transferases (BCATs) play crucial roles in the metabolism of branched-chain amino acids (BCAAs). The overexpression of *BCATs* promotes flowering in *Arabidopsis* by regulating the expression of genes regulating flowering time. *BCAT* can affect flowering in cucumbers [132]. Also, the overexpression of *CsBCATs* led to early flowering in *Arabidopsis*. *CsBCATs* can up-regulate the expression levels of *FT* and down-regulated the expression levels of *SOC1* in the GIGANTEA/CONSTANS (GI/CO) and SHORT VEGETATIVE PHASE (SVP)/FLC modules, thereby promoting early flowering in *Arabidopsis* [133].

*LFY* is a key gene influencing the development and flowering of plant floral organs [134]. It can also interact with genes such as *AGAMOUS* (*AG*) and *TERMINAL FLOWER 1* (*TFL1*) to regulate the onset of flowering [135]. *Cucumber-FLO-LFY* (*CFL*) in cucumber, which is homologous to *Arabidopsis LFY*, may participate in flowering [136]. The results of mRNA in situ hybridization indicate that the expression of *CFL* is related to the development of flower organs in cucumbers [137]. Transferring the *CFL* into gloxinia (*Sinningia speciosa*) for ectopic expression revealed that *CFL* could promote early flowering in gloxinia [138].

PHYB plays an essential role in regulating flowering in a photoperiod-dependent manner in *Arabidopsis* [139]; it degrades the factor promoting flowering, CO, to delay flowering [139,140,141]. A novel early flowering mutant was screened in cucumber. The mapping-based cloning revealed that *CsPHYB* (homolog of *Arabidopsis PHYB*) had a 5.5 kb long-terminal-repeat (LTR) retrotransposon insertion responsible for the mutation phenotype [48]. The ectopic expression of *CsPHYB* in *Arabidopsis* indicated that *CsPHYB* delayed flowering. Y2H and bimolecular fluorescence complementation (BiFC) assays showed that CsPHYB interacted with CsPIF3/4, promoting or inhibiting the expression of downstream flowering-related genes, thereby affecting flowering time.

*TFL1* is a mobile signal of the PEBP family, which controls *Arabidopsis* flowering [117,142,143]. Six PEBP family members have been reported in cucumber; *Csa3G776350* has the highest similarity to *AtTFL1*, reaching 71.91%, and is named *CsTFL1b* [113]. The ectopic expression of *CsTFL1b* in *Arabidopsis* showed that *CsTFL1b* delayed the flowering in *Arabidopsis*, and the degree of delayed flowering in transgenic plants was correlated with the expression level of *CsTFL1b* [144]. In addition, the *1-AMINOCYCLOPROPANE-1-CARBOXYLIC ACID SYNTHASE* (*CsACS2*) transferred into tobacco delayed flowering in tobacco [145]. Also, the heterotopic expression of *ETHYLENE RESPONSE FACTOR31* (*CsERF31*) also leads to late-flowering phenotypes in *Arabidopsis* and tobacco [146]. Despite abundant evidence, many mysteries regarding the molecular regulatory mechanisms of early flowering traits in cucumbers remain unresolved.

Researchers have identified a large number of early flowering germplasm resources for cucumbers. Several molecular markers have been designed to search for genes that can regulate the early flowering traits in cucumbers and elucidate their molecular regulatory mechanisms. These molecular markers can be used to detect the phenotype of cucumber flowering time in early development and even in the seedling stage [93], thus significantly improving breeding efficiency and providing the theoretical basis for promoting the research on early flowering research in cucumbers.

In conclusion, the molecular regulation of flowering in cucumbers involves a complex network of conserved pathways (photoperiod, gibberellin, etc.) and key genes (*CsFT*, *CsSPL13A*, *CsPHYB*, etc.), many of which exhibit functional homology with *Arabidopsis* flowering regulators. Significant progress has been achieved in characterizing these genes by exploring overexpression, protein–protein interactions (e.g., CsSHP-CsSEPs), and transposon-mediated mutations (e.g., CsPHYB-LTR), revealing their roles in flowering control. However, further studies are needed to elucidate the crosstalk between pathways and exploit these findings for precision breeding of early flowering cucumber varieties.

**Table 2:** Early flowering genes and their regulatory mechanisms in cucumbers.

Gene Names	Regulation	Interaction Proteins or TFs	References
*CsSPL13A*	positive	CsFT and CsBAM	[6]
*CsFT*	positive	-	[4,93,119,120]
*CsSHP*	positive	CsSEPs	[72]
*CsBCATs*	positive	FT and SOC1	[133]
*CFL*	positive	-	[138]
*CsMADS02*, *09*	positive	-	[129,130]
*CsMADS08*	positive	-	[131]
*CsPHYB*	Negative	CsPIF3/4	[48]
*CsTFL1b*	Negative	-	[144]
*CsACS2*	Negative	-	[145]
*CsERF31*	Negative	-	[146]

## 6. Functional Genomics and Omics Insights into Early Flowering Traits in Cucumbers

Cucumber genome data (CLv1.0) were released in 2009, making it the first sequenced genome of vegetable crops worldwide and laying a solid foundation for functional genomics research [147]. Subsequently, CLv2.0, CLv3.0, and CLv4.0 were reassembled, significantly advancing the progress of functional genomics research in cucumbers [148,149,150]. The cucumber genome data can be queried in cucumber-DB (http://www.cucumberdb.com/ (accessed on 22 March 2025)). This bioinformatics or genomic database can provide researchers with cucumber genome and transcriptome data, along with cucumber gene annotation, thereby providing a foundation for cucumber omics research. Thus, omics analyses, such as transcriptomic and proteomic analyses, are widely used to identify early flowering genes in cucumbers. Transcriptomic analysis is used for exploring gene evolution and function. New genes involved in cucumber flowering and their interacting factors were identified by integrating the transcriptional expression profiles and predicting promoter elements [151]. For example, RNA sequencing (RNA-seq) and differentially expressed gene analysis were conducted on male cucumber flowers, revealing that the HD-ZIP IV transcription factor *GL2-LIKE* regulated male flowering time in cucumbers [67]. RNA-seq analysis was conducted in the initiation stage of floral primordia and the developmental stage of floral organs to examine the differential expression of specific genes [27]. They found that *CsaNFYA1* integrated multiple types of genes to regulate the flowering of XIS cucumber. The transcriptome analysis was performed on plants of wild-type (“Hardwickei”) and cultivated (“9930”) cucumbers under SD and LD conditions. The results showed that the changes in a photoperiod-sensitive *CsFT* were associated with the day-neutral and early flowering of cultivated cucumbers [4]. The transcriptome analysis was conducted on “XIS49” cucumber under different light conditions. The results indicated that the *FT* gene, rather than its upstream circadian clock gene, regulated the SD flowering of XIS cucumber; also, the *cis*-regulation of *FT* might be due to TE insertion [120]. Proteomics research can be used to explore the mechanisms of cucumber flowering and the factors influencing it. Through proteomics research, Zhang et al. [138] demonstrated that CLF interacted with the LFY and MADS-box genes to regulate cucumber flowering time. A previous study showed that CsTFL1 competes with CsFT, interacts with CsNOT2a-CsFDP, and inhibits deterministic growth and terminal flower formation in cucumbers [152]. Moreover, *CsSPL3A* encoded a protein containing a nuclear localization signal and a highly conserved SBP domain, which, together with CsFT and CsBAM, mediated flowering in cucumbers [6].

In summary, the integration of functional genomics and multi-omics approaches has revolutionized the understanding of early flowering traits in cucumbers, from genome assembly to molecular networks. Transcriptomic studies have identified key flowering regulators such as *CsFT* and *CsaNFYA1*, whereas proteomic analyses have revealed critical protein interactions involving CsTFL1 and CsSPL3A. These findings highlight the power of omics technologies in uncovering both cis-regulatory mechanisms (e.g., TE-mediated *FT* regulation) and trans-acting factors governing flowering time. The established genomic resources and analytical frameworks provide a strong foundation for both fundamental research and precision breeding of early flowering cucumber varieties.

## 7. Challenges and Perspectives

**Figure 1:**
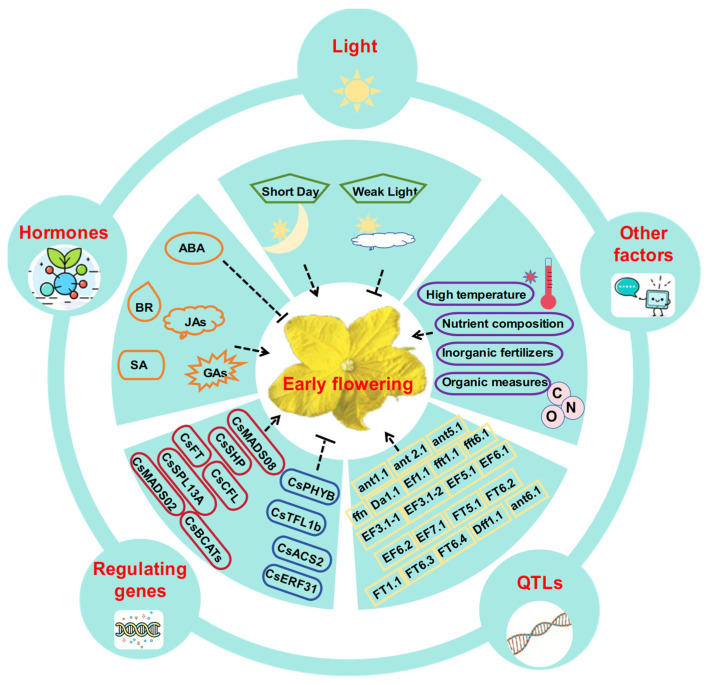
Factors regulating early flowering trait in cucumbers. The green boxes represent different light conditions, orange boxes indicate different hormones, purple boxes represent other factors, yellow boxes indicate QTLs, and red and blue boxes represent positive and negative regulatory genes, respectively. The dashed arrows represent the promotion of early flowering, whereas the dashed horizontal lines indicate the inhibition of early flowering.

Cucumber is an essential economic crop in China, with the highest yield and cultivation area worldwide. Early flowering is one of the main factors influencing critical agronomic traits such as cucumber growth period and yield. This is a key research direction in botany and an important goal for cucumber breeders. The phenomenon of early flowering in cucumbers is complex and is influenced by various factors such as temperature, light, plant hormones, and nutrients (Figure 1). Genetic research has clearly indicated that early flowering in cucumbers is a quantitative trait controlled by multiple genes. Numerous major and minor QTLs have been identified. Several genes have been identified as key regulators of early flowering in cucumbers. The development of high-precision, high-throughput phenotyping technology has significantly facilitated the investigation of these flowering traits. Furthermore, establishing comprehensive genetic resources, including overexpression libraries, mutant collections, and gene-edited populations, has provided both valuable germplasms and a solid theoretical foundation for advancing research on early flowering mechanisms in cucumbers.

Despite considerable advances in research on early flowering in cucumber, significant challenges persist due to the inherent complexity of this polygenic trait. The genetic architecture of early flowering involves intricate interactions among multiple genes and their dynamic interplay with environmental factors. This makes both phenotypic stability and gene identification particularly challenging. Environmental variability further complicates research outcomes by influencing trait expression. Moreover, technical limitations in genetic transformation and functional validation, including background-dependent effects in transgenic studies, hinder the achievement of consistent results. Translating laboratory findings into practical applications involves additional obstacles, particularly in implementing molecular breeding techniques and ensuring trait stability under commercial production conditions. Addressing these multifaceted challenges may require continued innovation in research methodologies and closer integration between molecular studies and breeding practices to advance both the fundamental understanding and agricultural applications of early flowering traits in cucumbers.

Currently, the identification of cucumber early flowering genes and research on molecular mechanisms in cucumbers mainly depend on transcriptomics and functional genomics, with relatively few applications of technologies such as proteomics, cytomics, and metabolomics. Therefore, future studies should focus on enriching the resources of early flowering cucumber varieties, actively conducting research on genetic laws, and fully using modern molecular biology techniques (e.g., gene editing and molecular marker-assisted selection breeding) to explore the genes related to early flowering in cucumber, thereby providing abundant genetic resources for the cultivation of early flowering cucumber varieties. Technologies such as genomics, transcriptomics, proteomics, metabolomics, and cytomics should be used to elucidate the mechanisms of early flowering in cucumbers. This would provide a theoretical basis for improving cucumber yield and quality, meeting market demand, and cultivating new early flowering varieties.

## Data Availability

All the data used in this review paper are available online.

## Abbreviations

The following abbreviations are used in this manuscript:

ABA	abscisic acid
BCAAs	branched-chain amino acids
BCATs	branched-chain amino acids and transferases
BiFC	bimolecular fluorescence complementation
BR	brassinosteroids
GAs	gibberellins
JA	jasmonic acid
LD	long-day
PAC	paclobutrazol
QTL	quantitative trait locus
RIL	recombinant inbred line
RNA-seq	RNA sequencing
SA	salicylic acid
SD	short-day
Y2H	yeast two-hybrid
